# Psychometric evaluation of the Persian version of Emergency Medical Services- Safety Attitudes Questionnaire (EMS-SAQ)

**DOI:** 10.1186/s12873-024-00941-y

**Published:** 2024-02-14

**Authors:** Roohangiz Norouzinia, Maryam Aghabarary, Pardis Rahmatpour

**Affiliations:** 1 Social Determinants of Health Research Center, Alborz University of Medical Sciences, Karaj, Iran; 2 School of Nursing, Alborz University of Medical Sciences, Karaj, Iran

**Keywords:** Emergency Medical Services, Patients Safety, Attitude

## Abstract

**Aim:**

This study aimed to conduct a psychometric evaluation of the Persian adaptation of the Emergency Medical Services Safety Attitudes Questionnaire (EMS-SAQ).

**Methods:**

In this cross-sectional study, the validity and reliability of the EMS-SAQ were assessed among 484 Iranian pre-hospital emergency department employees between February and June 2023.

**Results:**

Five factors were extracted namely safety climate, teamwork, job satisfaction, stress management, and working conditions with explained 38.75% of the total variance. The goodness of fit indexes confirmed the model (χ2 = 409.031, DF = 196, χ2 /df = 2.087, CFI = 0.900, IFI = 0.901, PCFI = 0.763 and PNFI = 0.701, and RMSEA = 0.069 [CI90% 0.059–0.078]).

**Conclusion:**

The Persian version of the SAQ-EMS, comprising 22 items across five factors, demonstrated good validity and reliability. It is recommended to undertake qualitative studies focusing on the concept of patient safety in pre-hospital settings, considering diverse contexts and cultural nuances to develop more robust assessment tools.

## Introduction

Patient safety stands as a pivotal component influencing the quality of healthcare services [1]. The safety culture within any healthcare institution reflects the beliefs and perceptions of its employees regarding organizational safety standards and the well-being of those within the system [2]. The impact of workplace attitudes and beliefs on patient care safety cannot be understated [3].The realm of patient safety within healthcare systems remains a sensitive and critical concern [4]. Efforts aimed at its enhancement play a significant role in reducing accidents and injuries resulting from patient care practices [5]. Among the dimensions of public culture, patient safety culture has emerged as a top priority for healthcare facilities. Its essence lies in the prevention, improvement, and rectification of adverse events stemming from the healthcare delivery process [6].

Research findings reveal that a concerning percentage of hospital-admitted patients—around 3–17%—experience preventable injuries or complications. These incidents highlight the necessity for healthcare facilities to adopt straightforward yet effective methods to enhance patient safety [7]. Establishing a comprehensive view of patient safety is vital for medical centers to leverage these methods effectively.

A robust safety culture within medical centers heavily relies on various factors: the perception of the center’s commitment by its employees, error reporting and subsequent learning, enhancement of patient safety levels, fostering teamwork, and recognizing and encouraging employees who prioritize these aspects [8].

Limited studies have investigated workplace safety culture in the pre-hospital area. The pre-hospital team provides services outside the hospital in a high-stress environment. Examples of these threats to patient safety include falling off the stretcher carrying the patient, errors in placing the endotracheal tube, misdiagnosis of signs and symptoms, and deviation from the standard treatment protocol [2, 9].

Other threats include poor understanding of the connections between jobs related to the health field, unfavorable management, and an incomplete understanding of specific patient groups [2, 10]. In addition to airway management errors that are common and dangerous, other studies have pointed to equipment malfunctions, ambulance accidents, improper patient handling, medical mismanagement, and the non-implementation of existing protocols and guidelines [11].

On the other hand, emergency employees are often exposed to high stress and burnout [12] and there are also concerns about the accuracy of care decisions. Hence, according to the observations and studies, it can be concluded that the pre-hospital emergency workplace culture can affect patient safety [13].

By examining the views and attitudes of health workers and their performance towards the culture of patient safety, it is possible to identify treatment errors in the treatment fields that threaten patient safety and try to eliminate or minimize them, which causes providing high-quality services to patients is, in fact, the ultimate goal of all centers and fields of treatment and health care [14]. Therefore, a suitable tool is needed to measure this concept. There are some safety culture instruments, namely the Hospital Survey on Patient Safety Culture (HSPSC) [15], the Manchester Patient Safety Framework [16] and the Safety Attitudes(SAQ) [10]. The SAQ has been validated in several countries and populations [17–20]. In 2010, Patterson modified the SAQ and developed a new scale entitled “emergency medical services safety attitudes’ questionnaire (EMS-SAQ)”, especially for emergency medical Services (EMS) employees [2]. Due to the fact that there was no tool in Persian to examine the patient safety culture in the pre-hospital area, this study was designed with the aim of translating and psychometrically analyzing the EMS-SAQ among Iranian EMS employees.

## Methods

### Design

This cross-sectional study was conducted to evaluate the psychometric properties of the Persian version of the EMS-SAQ among 484 Iranian employees of the pre-hospital emergency department from February to June 2023. An online survey was created using the Persian online questionnaire platform (www.porsline.ir) and data was collected by sending the link to the questionnaire to EMS employees in Telegram, What’s App, or email. The inclusion criteria in this study were the operational employees of EMS who were willing to be part of this study. Sample selection was based on convenience sampling.

### Measurements

The online questionnaire consists of two parts: (1) demographic and job information (i.e., age, gender, educational level, year of work, and job position); and (2) the Persian version of EMS-SAQ.

The EMS-SAQ comprises 30 main items that are placed in 6 dimensions: safety climate (3-4-8-9-14-15-22); teamwork climate (1-6-11-23-31); perceptions of management (7-12-13-19); job satisfaction (2-17-24-27-28-29); working conditions (5-16-32-44); and stress recognition (18-25-26-34). In addition, 20 questions have been included by the developer according to the working conditions of EMS staff. All answers are on a 5-point Likert scale from strongly agreeing (4) to strongly disagreeing (0) [2]. In this study, the analysis was conducted only on the main items.

### Translation

After obtaining permission from the developer of the EMS-SAQ, the forward-backward translation technique according to the World Health Organization was used to translate the EMS-SAQ to Persian. Two translators translate it from English to Persian independently. Then, we integrated the two sets of Persian versions of EMS-SAQ into one and provided it to a Persian-English translator to translate it back into English. Next, the English version sent to the one expert in the field of study was reviewed to confirm the originality and accuracy of the translated measure.

### Validity assessment of the Persian version of the EMS-SAQ

The face, content, and construct validity of the Persian version of EMS-SAQ were evaluated. Fifteen pre-hospital employees were asked to assess the difficulty and ambiguity as a face validity step. According to the participants’ viewpoints, necessary corrections were made for some items. Then, to achieve content validity, 15 faculty members in the fields of nursing, medical emergencies, and health in emergencies and disasters were requested to assess the item’s necessity and relevancy by content validity ratio (CVR) and modified kappa coefficient (K), respectively. Following the recommendation by Lawshe the minimum value for CVR should be 0.49 for ten experts [21]. Finally, each item’s modified kappa coefficient (K) was obtained, with its minimum value of 0.60 or above, to establish each item’s content validity [22].

To assess construct validity, the maximum likelihood EFA with Promax rotation was performed on the first dataset (*n* = 252) to extract the factor structure of the Persian version of EMS-SAQ. The Kaiser-Meyer-Olkin (KMO) test (> 0.5 is good) [23], along with Bartlett’s test of sphericity, was subsequently applied to determine sampling adequacy as well as the appropriateness of the data for factor analysis. To extract factorial structure, this study follows the criteria of (1) eigenvalues of more than 1; (2) commonalities of more than 0.3, and (3) indication of scree plots [24]. Also, items with a factor loading of less than 0.4 were removed.

For confirming the factor structures obtained after EFA analysis, the CFA performed on second dataset (*n* = 232) by using common model fit indices included the Chi-square test (χ2), the minimum discrepancy function by degree of freedom (CMIN/DF) < 3, comparative fit index (CFI) > 0.90, incremental fit index (IFI) > 0.90, parsimonious comparative fit index (PCFI) > 0.5, parsimonious normed fit index (PNFI) > 0.5, and root mean square error of approximation (RMSEA) < 0.08 [22].

### Reliability assessment of the Persian version of EMS-SAQ

Cronbach’s alpha (α) and McDonald’s omega (Ω) were assessed for internal consistency, and composite reliability (CR) and maximum reliability (Max H reliability) were evaluated for reliability of scale. The values > 0.7 were considered acceptable for all these indices.

### Ethical consideration

The protocol of this study was approved by the ethical committee of Alborz University of Medical Sciences [Ethic code: IR.ABZUMS.REC.1401.295]. The study aim, information about items, voluntary participation, the possibility of withdrawing from the study at any time, and confidentiality and anonymity of information were inserted on the first page of the online questionnaire. In this study, informed consent was obtained online.

## Results

The mean age of 484 EMS employees were 22 years (SD = 5.7), and most of them (65.3%) were married. The mean years of their work experiences were 8.87 ± 5.1 years.

**Fig. 1:**
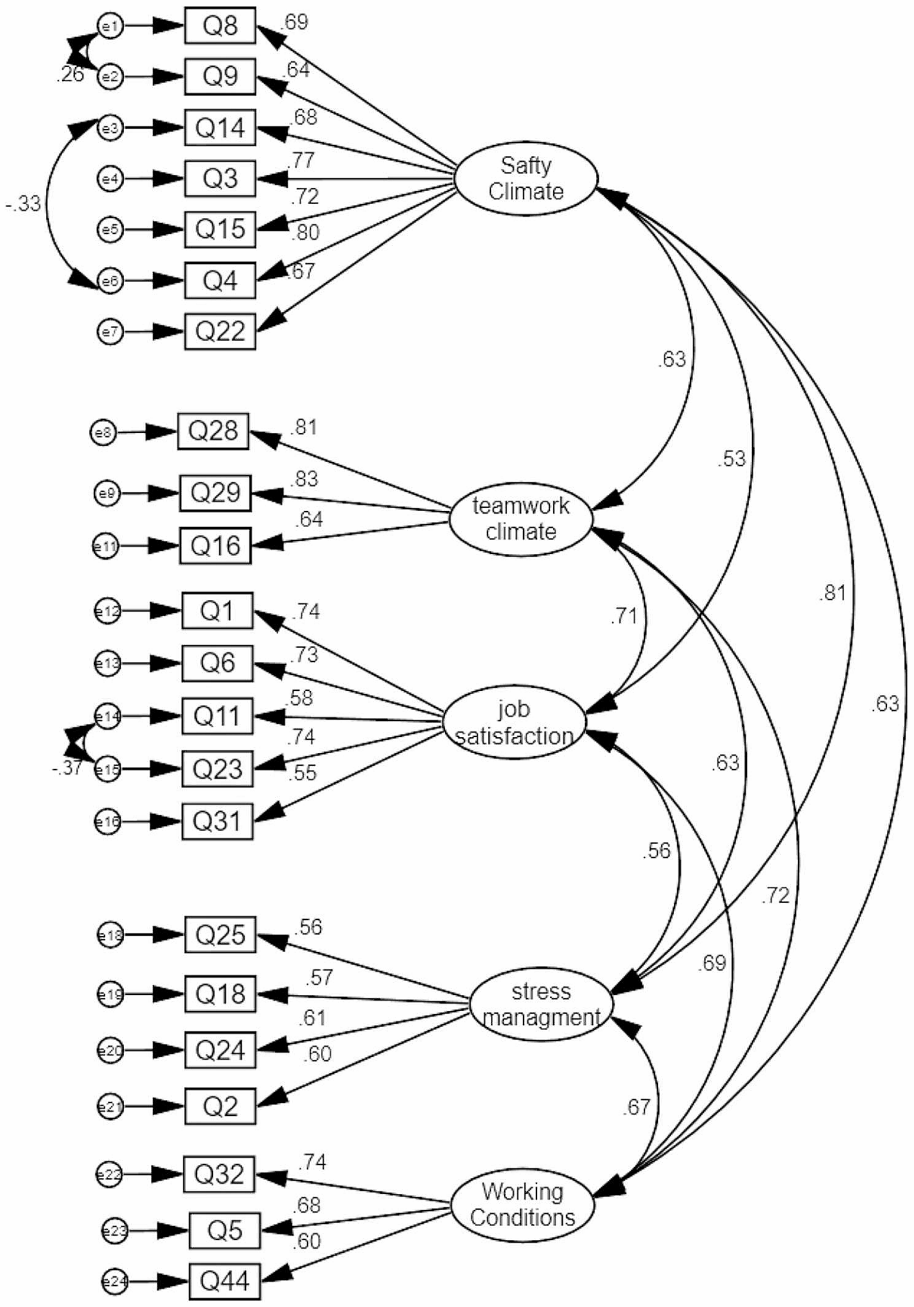
The structure model of the Persian version of EMS-SAQ

The KMO (0.901) and Bartlett test (2456.435, df = 276, *p* < 0.001) showed that the strength of the partial correlation between the variables is suited to EFA. Based on the EFA results, 24 items in five factors were determined: safety climate, teamwork, job satisfaction, stress management, and working conditions (Table 1). These factors explained 38.75% of the total variance of the Persian version of the EMS-SAQ among the EMS employees. After conducting CFA, two items (27 and 34) below 0.5 was deleted, so the final scale has 22 items. The fit indices of the CFA model showed that χ2 = 409.031, DF = 196, χ2 /df = 2.087, CFI = 0.900, IFI = 0.901, PCFI = 0.763 and PNFI = 0.701, and RMSEA = 0.069 [CI90% 0.059–0.078] (see Fig. 1).

**Table 1:** The EFA and reliability results of Persian version of EMS-SAQ

EFA						Reliability			
factors	Items	Factor loading	h2 *	λ^**^	Variance (%)	Cronbach’s alpha	McDonald’s omega	CR	MaxR(H)
Safety climate	Q8	0.835	0.579	3.1	12.91	0.785	0.73	0.804	0.818
	Q9	0.803	0.521						
	Q14	0.726	0.567						
	Q3	0.653	0.597						
	Q15	0.535	0.479						
	Q4	0.534	0.594						
	Q22	0.443	0.569						
Teamwork climate	Q28	0.903	0.693	1.95	8.12	0.751	0.88	0.808	0.831
	Q29	0.877	0.727						
	Q27	0.447	0.277						
	Q16	0.311	0.462						
Job satisfaction	Q1	0.825	0.639	1.8	7.5	0.764	0.79	0.877	0.882
	Q6	0.584	0.533						
	Q11	0.533	0.348						
	Q23	0.524	0.509						
	Q31	0.415	0.413						
Stress Management	Q34	0.740	0.386	1.45	6.04	0.714	0.77	0.710	0.720
	Q25	0.583	0.431						
	Q18	0.530	0.383						
	Q24	0.350	0.376						
	Q2	0.336	0.299						
working conditions	Q32	0.651	0.598	1	4.16	0.700	0.86	0.700	0.712
	Q5	0.579	0.521						
	Q44	0.464	0.460						

* h^2^, item communality,**λ, eigenvalue, Total variance: 38.75%

Based on the indices shown in Table 1, the Persian version of EMS-SAQ has good internal consistency and reliability.

## Discussion

The EMS personnel, being the initial responders to various incidents, have a responsibility to deliver quality services promptly and in line with scientific guidelines. They consistently encounter significant work pressure and confront numerous challenges, including insufficient staff, managerial problems, equipment shortages, and a lack of support [25].

EMS personnel face a dynamic and challenging environment in pre-hospital emergency care, which increases the likelihood of care and treatment errors [26]. The factors influencing adverse events in this setting are largely unknown [27], leading to an ambiguous safety situation [28]. Limited research has been conducted on patient safety in pre-hospital emergency care, resulting in a lack of understanding in this area [29]. Evaluating the understanding and attitude of all staff towards patient safety is crucial for establishing and improving the safety culture [30]. Patient safety culture is a significant predictor of adverse events, and enhancing it can reduce their occurrence. Therefore, healthcare organizations should regularly assess and enhance their safety culture to prevent unintentional harm to patients [31]. The safety culture in pre-hospital emergencies varies across different regions. It is not logical to apply the findings of studies conducted in one country to other countries, as each country has its own unique culture and moral values [32].

This study was the first psychometric evaluation of the Persian version of EMS-SAQ. Although this questionnaire was translated and used in the cross-sectional descriptive study of Najafi Ghezeljeh et al., the psychometric characteristics were not evaluated [32]. The original questionnaire had 30 main items in six factors, which the present study does not confirm. Therefore, the EFA was conducted, and six items (items 7, 12, 13, 17, 19, and 26) were omitted at this stage and 2 items (27 and 34) were removed in CFA stage.

In the present study, the first factor extracted was “safety climate” (7 items), with the highest percentage of total explained variance (12.91%). This factor addressed the understanding of organizational commitment to safety and how to deal with errors. Several common interventions that EMS agencies should consider improving safety include an error-reporting system without criticism, providing a program to learn how to report errors and learn from errors, and medication safety feedback forms [2].

The second extracted factor was “teamwork climate” (3 items), with 8.12% of the total explained variance, which referred to employees’ understanding of cooperation between staff and working as a team. In this factor, some changes were made compared to the original questionnaire. Some new items were placed (items 2, 24, and 16), and one item was deleted (item 17). These changes may be due to the cultural differences and working and organizational conditions in Iran, or they may be related to the way the participants answered the questions in the questionnaire. The results of Alsabri et al. study revealed that teamwork and communication skills training can affect safety culture and patient outcomes [33]. They suggested these interventions to reduce the incidence of medical errors and adverse events.

The third extracted factor was labeled “job satisfaction” (7.5% of the total variance). It comprised five items reflecting the issue of job and workplace satisfaction. This factor, as the “safety climate” factor, was completely consistent with the original questionnaire. Labrague et al. stated that the work environment affects quality of care, adverse events, and job satisfaction [34]. Therefore, enhancing nurse work environments to improve job satisfaction and patient safety outcomes as a potential strategy can be suggested. According to Rashtchi et al., it is advantageous to modify reward systems and implement performance improvement strategies in the prehospital emergency department to increase motivation and improve staff performance [35]. Implementing these measures can effectively contribute to enhancing job satisfaction.

The fourth extracted factor was “stress management” with 4 items, and 6.04% of the total variance. In this factor, item 26 was removed and two items from “safety climate” factor were placed in this factor. Due to these changes, the name of this factor was changed to “stress management”. In this factor, the items deal with the stress of the employees, and how the management and colleagues deal with each other. Stress may affect the performance which without a doubt important to establish a safety culture [36].

The last factor was labeled “working conditions.” It has 3 items and 4.16% of the total explained variance. This factor referred to the perceived quality of the work environment and logistical support (such as staff and equipment). Working conditions influence patient outcomes and staff’s job satisfaction. In better working conditions, health workers can provide safer and more standard patient care than in poorer work environments [34].

The CFA confirmed the good fit of the model. Furthermore, all extracted factors of the Persian version of the EMS-SAQ demonstrated acceptable reliability and internal consistency (α >. 70 & Ω >. 73). In the study of Venesoja et al. which was conducted to validate the EMS-SAQ, the results of the CFA showed that the model did not fit perfectly with the data collected from Finnish EMS workers and needed to be adjusted [37]. Otherwise, reliability scores were acceptable in the study. Therefore, the authors stated that despite the results of the CFA, which were not entirely favorable, the Finnish version of the questionnaire can be used in Finland. In a study conducted in 2016 to validate the Dutch version of the SAQ (NL), the results showed that the initial model was fit, and the questionnaire had good internal consistency and reliability. The respondents in this study included doctors and nurses from different hospital departments, and academic and non-academic hospitals [38]. Also, the results of Zimmermann et al. study showed that the German version of the SAQ had acceptable psychometric properties [19]. However, the content validity results and the non-response of numerous participants to several items, especially in the “perceptions of management” factor, showed that re-translation and adjustment in these items are needed. In this case, it is necessary to re-evaluate the characteristics of psychometrics in hospitals and different departments with random samples after these revisions.

Another study was conducted in Norway in 2014 to validate the SAQ tool. In the CFA, five factors, including “teamwork climate”, “safety climate”, “job satisfaction”, “working conditions”, and “perceptions of management,” wereconfirmed.The respondents to this study comprised doctors and nurses. In this study, an item was transferred from the “perceptions of management” factor to the “working conditions” factor. Also, the “stress recognition” factor was completely removed. The authors stated that several studies showed that the “stress recognition” factor is not valid as an organizational climate scale. The CFA showed that the questionnaire, which included five factors, had good fit indices. Cronbach’s alpha ranged from 0.67 to 0.83 for different factors [39].

In another study, which examined the psychometric properties of the Georgian version of the EMS-SAQ, the results showed that the original model of the questionnaire did not fit. Therefore, construct validity was done and four factors emerged. These factors included “job satisfaction and safety climate”; “teamwork climate”; “stress recognition”; and the “perceptions of Hospital Management” that the items related to working conditions are also included. The internal consistency of the instrument was confirmed by Cronbach’s alpha coefficient index from 0.61 to 0.91 [36]. Several studies in other countries revealed some variation in SAQ’s CFA results that may be related to cultural differences. They stated that cultural differences could affect an organization’s safety culture [40–42].

### Limitations

The sample was recruited from Iranian EMS employees; therefore, the generalizability of the findings is limited. Despite the advantages of using an online questionnaire, the lack of physical interaction, the inability to verify an individual’s status, and the veracity of their responses were limitations of this online survey. Also, social desirability bias, which can result from the use of Likert scales in surveys, could cause respondents to choose answers that do not accurately reflect their actual experiences or opinions.

### Implications

Understanding the nuanced aspects of safety culture among EMS personnel allows for the development of tailored interventions and training programs. These could address specific challenges faced in the local context, such as error reporting systems, training on error management, and fostering a culture of open communication. The identification of cultural differences influencing safety perceptions highlights the necessity for adapting safety protocols and interventions to suit the unique cultural and organizational conditions present in Iran or similar contexts.

## Conclusion

The Persian version of the SAQ-EMS questionnaire, with 22 items in five factors including “safety climate”, “teamwork”, “job satisfaction”, “stress management”, and “working conditions,” has good validity and reliability. This questionnaire is self-reported, so it has all the weaknesses and limitations of self-reported questionnaires. The recommendation is to conduct qualitative studies on the concept of patient safety in pre-hospital settings, considering various contexts and cultures, in order to create more reliable instruments.

## Data Availability

The data that support the findings of this study are available on request from the corresponding author.
